# Optimizing the Extraction and Enrichment of Luteolin from *Patrinia villosa* and Its Anti-Pseudorabies Virus Activity

**DOI:** 10.3390/molecules28135005

**Published:** 2023-06-26

**Authors:** Lian Fu, Su Li, Xiaoyu Men, Xiaojing Cai, Zhiying Wang, Yongkang Xu, Zhiyuan Ren, Yi Shao, Yan Zhu

**Affiliations:** 1 College of Veterinary Medicine, Northeast Agricultural University, Harbin 150038, China; a1473704626@163.com (L.F.); mxy350925@163.com (X.M.); caixiaojing777@126.com (X.C.); wzyzy0419@163.com (Z.W.); 18754963560@163.com (Y.X.); zhiyuanren0328@163.com (Z.R.); sy15049522319@163.com (Y.S.); 2 College of Resources and Environment, Northeast Agricultural University, Harbin 150038, China; lisu@caas.cn

**Keywords:** *Patrinia villosa*, luteolin, response surface methodology, macroporous resin method, antiviral activity

## Abstract

Luteolin from *Patrinia villosa* exhibits strong antiviral activity. Here, the conditions for extracting and enriching luteolin from *P. villosa* were optimized. Response surface methodology was used to determine the optimal extraction parameters in terms of reflux time, solvent ratio, extraction temperature, material-to-liquid ratio, and number of extractions. Thereafter, a macroporous resin method was used to enrich luteolin from *P. villosa*. Finally, the following optimal extraction and enrichment conditions were established: an extraction time of 43.00 min, a methanol/hydrochloric acid solvent ratio of 13:1, an extraction temperature of 77.60 °C, a material/liquid ratio of 1:22, and a total of two extractions. NKA-9 was determined to be the most appropriate resin for enrichment. The ideal adsorption conditions were as follows: a pH of 5.0, a temperature of 25 °C, an initial luteolin concentration of 19.58 µg/mL, a sample loading volume of 2.9 BV, and a sample loading rate of 2 BV/h. The ideal desorption conditions were as follows: distilled water, 30% ethanol and 80% ethanol elution, and 5 BV at a flow rate of 2 BV/h. After optimization, the enrichment recovery rate was 80.06% and the luteolin content increased 3.8-fold. Additionally, the enriched product exhibited a significant inhibitory effect on PRV (Porcine pseudorabies virus) in vitro and in vivo, providing data for developing and applying luteolin from *P. villosa*.

## 1. Introduction

*Patrinia villosa* is a traditional Chinese medicine [1] that exhibits good dehumidification, detoxification, stasis, and pain relief effects. This medicine is often used to treat dry heat, constipation, enteritis [2,3], postpartum blood stasis, intestinal carbuncles, furuncles [4], swelling, and other symptoms as well as lung abscesses, hepatitis, tumors, and other diseases. The components of *P. villosa* are complex and include flavonoids [5], terpenoids, steroids, polyphenols, saponins, polysaccharides, and other active substances [6,7,8]. The flavonoid luteolin is present in high amounts in *P. villosa* and exhibits antiviral, antioxidant, anti-inflammatory, and nervous-system-protective effects and improves cognitive function. Luteolin exhibits antitumor effects by inhibiting tumor cell proliferation and promoting apoptosis [9,10]. Studies have shown that the 3,4-OH and 5-OH groups of luteolin are good for antiviral activity [11]. Therefore, optimizing the processes of extracting and enriching luteolin is very valuable for its further development.

Luteolin, as an important natural yellow fermentation compound, widely exists in various medicinal plants, fruits, and vegetables. The name of luteolin comes from the leaves, ganoderma, and branches of the plants of the Genius family. The melting point of luteolin is 328–330 °C, and the boiling point is 616 °C. From other structures, it can be seen that it is a chemical substance with various fragrances, with poor lipophilicity and hydrophilicity. Due to the structure of luteolin, its solubility in general solvents is relatively poor, making the manufacturing equipment of luteolin difficult. Luteolin has a wide range of biological activities. It can fight against viruses, inflammation, and small bacteria; reduce the damage caused by the excessive synthesis of reactive oxygen species; and also solve nervous system diseases. It can also help people improve their memory and cognitive abilities. The anticancer effect of luteolin prevents the growth of tumor cells and promotes their death.

At present, the methods of extracting luteolin generally include hot reflux extraction, ultrasonic extraction, microwave-assisted extraction, and supercritical extraction. The advantages of the hot reflux extraction method are its simple and safe operational procedures, high extraction efficiency, and low cost of consumables. Therefore, this method is widely used in industrial production. Compared with the high-temperature boiling method, refluxing can more quickly and effectively improve the diffusion and dissolution rates of soluble components and promote the faster dissolution of target components. Ultrasonic extraction uses an ultrasound-assisted solvent. The target components in plant cells are damaged due to the bubble cavitation effect and agitation caused by the strong ultrasound vibrations, which promotes their release into the solvent [12]. Ultrasonic extraction is advantageous because it can shorten the extraction time and improve the extraction rate. Microwave-assisted extraction is a new method that combines the microwave and solvent extraction methods. Microwave-assisted extraction exhibits several advantages, including high extraction efficiency, simple equipment, and low pollution; thus, the method is widely used. Supercritical extraction is a green extraction technology because it does not require the use of organic solvents. This method exhibits several advantages, including being nontoxic, harmless, and simple to perform; thus, supercritical extraction is often used in the food and medical industries [13]. At present, many studies have reported the enrichment and purification of luteolin from different plant sources by macroporous resin methods. Wang Gang [14] and others used a macroporous resin method to enrich luteolin from honeysuckle, others used this method to enrich luteolin from poplar flowers, and Song Xinrui [15] and others used this method to enrich luteolin from dandelions. Many studies have shown that enriching and purifying luteolin from plants by macroporous resins is feasible. Luteolin, an important flavonoid in *P. villosa*, exhibits extensive pharmacological effects and biological activities. To find scientific methods to fully utilize and develop the luteolin found in *P. villosa*, the processes of extracting and enriching luteolin must be established and optimized. At present, there is no reported research on the application of the hot reflux extraction method to extract and enrich luteolin from *P. villosa*. Therefore, this study used a single-factor approach to optimize the luteolin extraction process by response surface methodology, optimized the enrichment process by the macroporous resin method, and evaluated whether the enriched product shows anti-pseudorabies virus (PRV) activity, providing a reference for the further utilization and development of *P. villosa* and luteolin.

## 2. Results

### 2.1. Effect of Extraction Time on the Luteolin Extraction Rate

**Figure 1:**
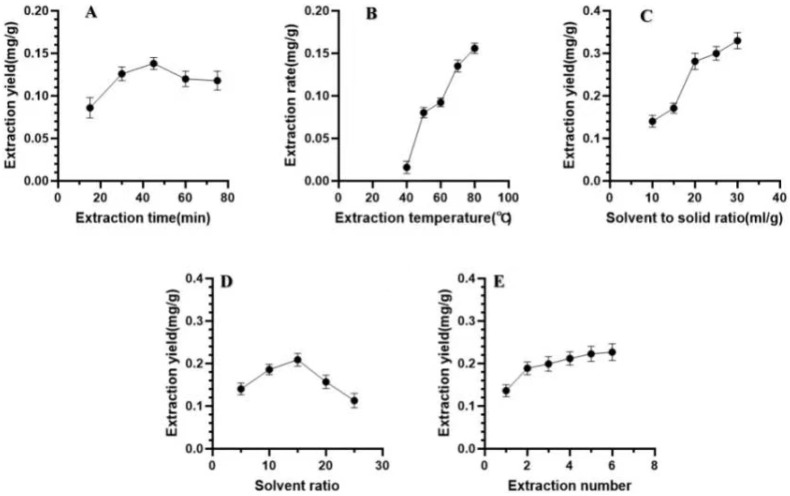
Effects of different extraction parameters on the extraction yield of luteolin. Single-factor experiments were conducted by varying the extraction time, extraction temperature, material/liquid ratio, methanol/hydrochloric acid solvent ratio, and number of extraction times to study the influence of these five factors on the extraction rate of luteolin. (**A**), the effect of extraction time on the extraction rate of Luteolin; (**B**), the effect of extraction temperature on the ex-traction rate of Luteolin; (**C**), the effect of liquid to material ratio on the extraction rate of Luteolin; (**D**), the influ-ence of solvent ratio (methanol: hydrochloric acid) on the extraction rate of Luteolin; (**E**) shows the effect of extrac-tion frequency on extraction rate.

As the extraction time is extended, the contact time between the target component and the solvent becomes longer, and the extraction rate increases. However, if the extraction time is too long, some target compounds will be degraded, and the extraction rate decreases; this will also increase the consumption of time and energy. In this study, the ratio of liquid to material was fixed at 20 mL/g, the ratio of methanol to hydrochloric acid was fixed at 20:1, the reflux temperature was fixed at 70 °C, and the number of extractions was fixed at 1 to assess the effect of extraction (reflux) time on the extraction rate of luteolin from *P. villosa*. The reflux times were 15, 30, 45, 60, and 75 min. These results are shown in Figure 1A. The extraction rate of luteolin increased when the time increased from 15 min to 45 min and then decreased with increasing extraction time. There was a significant difference between the extraction times of 30 min and 15 min, so the center point of the orthogonal experiment was 30 min.

### 2.2. Effect of Extraction Temperature on the Luteolin Extraction Rate

An increase in extraction temperature is not only conducive to mutual movement between the solute and solvent molecules, but also decreases the viscosity of the organic solvent, thus increasing the extraction rate. However, some thermosensitive compounds exhibit higher losses due to excessive temperature, which affects their stability and reduces the extraction rate. In this study, the ratio of material to liquid was fixed at 1:20, the ratio of hydrochloric acid to methanol was fixed at 1:20, the extraction time was fixed at 45 min, and the number of extractions was fixed at one to study the influence of extraction temperature on the rate of luteolin extraction from *P. villosa*. The extraction temperature was set to 40, 50, 60, 70, and 80 °C. These results are shown in Figure 1B. The extraction rate of luteolin increased with increasing reflux temperature. There was a significant difference between the extraction rate of luteolin at 70 °C and that at 40–60 °C. Therefore, the center point of the extraction temperature orthogonal experiment was 70 °C.

### 2.3. Effect of Material/Liquid Ratio on the Luteolin Extraction Rate

The equilibrium constant and solubility of the target compound are affected by the material-to-liquid ratio [16], so this ratio cannot be ignored during the extraction process. A liquid-to-material ratio that is too high will increase experimental consumables and lead to excess costs, and a liquid-to-material ratio that is too small will lead to insufficient extraction of the target compounds. Therefore, it is very important to select an appropriate material/liquid ratio [17]. These results are shown in Figure 1C. The effect of the material/liquid ratio on the rate of luteolin extraction from *P. villosa* was investigated under the constant conditions of 45 min of reflux time, reflux temperature of 70 °C, a 20:1 methanol/hydrochloric acid ratio, and one extraction, and the material/liquid ratio was set to 10 mL/g, 15 mL/g, 20 mL/g, 25 mL/g, and 30 mL/g. These results are shown in Figure 1C. The extraction rate of luteolin increased when the material/liquid ratio increased from 10 mL/g to 15 mL/g, but the difference was not significant. When the liquid-to-material ratio was 20 mL/g, a significant difference was observed. Therefore, the center point of the liquid-to-material ratio orthogonal experiment was 20 mL/g.

### 2.4. Effect of Methanol/Hydrochloric Acid Solvent Ratio on the Luteolin Extraction Rate

Luteolin is very soluble in methanol [18], and the extraction solvent used for reflux extraction in this study was a hydrochloric acid–methanol mixed solution. However, when the concentration of hydrochloric acid was too high, the extraction rate of luteolin did not increase, and too strong of an acid causes damage to the chromatographic column and injection valve. Under constant conditions, i.e., a reflux temperature of 70 °C, material/liquid ratio of 1:20, extraction time of 45 min, and one extraction, the influence of the ratio of methanol to hydrochloric acid in the extraction solvent on the extraction rate was studied. The methanol/hydrochloric acid ratio was set to 5:1, 10:1, 15:1, 20:1, and 25:1. These results are shown in Figure 1D. At a ratio of 15:1, the extraction rate of luteolin was the largest, with a significant difference from the results obtained at ratios of 10:1, 20:1, and 25:1. Therefore, it was determined that 1:15 was the center point of the experiment.

### 2.5. Effect of the Number of Extractions on the Luteolin Extraction Rate

In the extraction process, the production efficiency is related to the energy consumption and the number of extractions, so selecting an appropriate number of extractions will reduce the cost. Under the above optimal conditions, the extraction rate of luteolin was studied, and the number of extractions was set to 1, 2, 3, 4, 5, and 6. These results are shown in Figure 1E. The extraction rate of luteolin increased significantly when the number of extractions was increased from one to two, but the increase was not significant after two extractions. Finally, it was determined that it was best to extract luteolin twice.

### 2.6. Results of Response Surface Optimization

#### 2.6.1. Model Fitting and Statistical Analysis

On the basis of the above single-factor experimental analysis, a Box–Behnken design (BBD) response surface approach was used to design an experiment considering the following variables: material/liquid ratio, reflux time, reflux temperature, and methanol/hydrochloric acid solvent ratio. The effects of these variables and their effects on the extraction rate of luteolin were explored. These results are shown in Table 1 and Table 2, which include the designed experimental conditions, results, and ANOVA results for the model. The following equation can be used to express the relationship of each variable to the response value: Y = 0.61423 + 1.55889 × 10^−3^X_1_ − 8.86500 × 10^−3^X_2_ − 1.47992 × 10^−2^X_3_ + 1.508500 × 10^−2^X_4_ + 8.96667 × 10^−5^X_1_X_2_ + 6.49970 × 10^−6^X_1_X_3_ − 8.53333 × 10^−5^X_1_X_4_ + 1.7 × 10^−5^X_2_X_3_ + 2.37 × 10^−4^X_2_X_4_ − 3.34 × 10^−4^X_3_X_4_ + 8.09259 × 10^−6^X_12_ + 9.883333 × 10^−5^X_22_ + 1.31083 × 10^−4^X_32_ + 1.56333 × 10^−4^X_42_. ANOVA was used to determine whether the experimental model was significant. See Table 2 for these results. The value of *p* represents both the significance of each factor and the interaction between each parameter. *p* was <0.0001 in this study, indicating that this model is significant. The determination coefficient (R^2^) was 0.9545, and the adjusted retention coefficient (Adj-R^2^) was 0.9014, both of which are close to 1; thus, this model was applicable to this experiment. The F value of the mismatch term in the model indicates whether the experimental data fit the model regression. The F value in this study was 13.93, which indicates that this model is valid.

#### 2.6.2. Interaction Results

**Figure 2:**
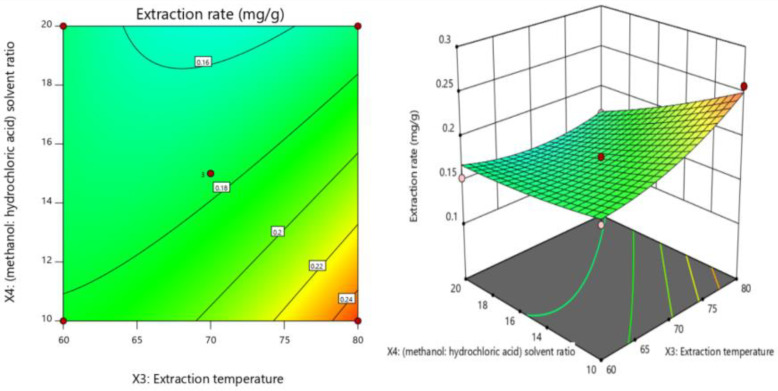
Contour map (2D) and response surface graph (3D) of the effect of extraction temperature and solvent concentration ratio on response value Y. The interaction effect between the extraction temperature (X_3_) and methanol/hydrochloric acid solvent ratio (X_4_) (X_3_X_4_) was the most significant. With increasing extraction temperature (X_3_) and decreasing methanol/hydrochloric acid solvent ratio (X_4_), the extraction rate of luteolin increased.

The influence of the interaction between any two factors on the extraction rate of luteolin can be directly visualized through a two-dimensional contour map and a three-dimensional response surface map. The significance of the variable relationship is reflected by the two-dimensional contour map, and the sensitivity of the response value to changes in variables is reflected by the three-dimensional response surface map [19]. The interaction between the extraction temperature (X_3_) and methanol/hydrochloric acid solvent ratio (X_4_) (X_3_X_4_) is shown in Figure 2. This interaction showed the most significant effect on the extraction rate in this study. With increasing extraction temperature (X_3_) and decreasing methanol/hydrochloric acid solvent ratio (X_4_), the extraction rate of luteolin increased. The interactions between the other factors did not have a significant effect on the extraction rate of luteolin, so these results are not shown.

#### 2.6.3. Extraction Parameter Optimization and Model Validation

As shown in Table 3, the experimental value was 0.265 ± 0.03 mg/g, which is close to the predicted value and higher than the value under nonoptimal conditions, indicating that this model can be used to extract luteolin from *P. villosa*.

### 2.7. Enrichment of Luteolin from P. villosa by Macroporous Resin

#### 2.7.1. Screening of the Macroporous Resin

**Figure 3:**
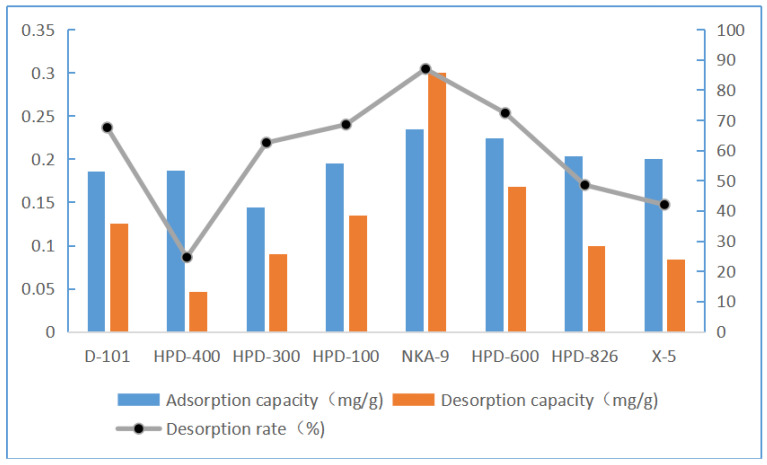
Static adsorption and desorption capacities and desorption rates of eight resins for luteolin. Resins NKA-9, HPD-600, X-5, and HPD-826 showed good adsorption capacity. NKA-9 resin had the best desorption capacity, while X-5 and HPD-826 exhibited poor desorption capacity. Therefore, only NKA-9 resin showed both good adsorption capacity and strong desorption capacity. The adsorption capacity of macroporous NKA-9 resin for luteolin was 0.24 mg/g, and the desorption rate was 86.90%. Thus, NKA-9 was selected for the adsorption kinetics experiment.

These results are shown in Figure 3. Among the eight macroporous resins, NKA-9, HPD-600, X-5, and HPD-826 exhibited good adsorption capacities. Resin NKA-9 showed the best desorption capacity, while X-5 and HPD-826 exhibited a poor desorption capacity. Therefore, only NKA-9 resin showed a good adsorption capacity and a strong desorption capacity. The adsorption capacity of the macroporous NKA-9 resin for luteolin was 0.24 mg/g, and the desorption rate was 86.90%. Finally, NKA-9 was selected for the adsorption kinetics experiment.

#### 2.7.2. Effect of pH Value on Adsorption Capacity

The concentration of hydrogen ions has an effect during the resin adsorption process because changes in the pH value and the ionization degree of the molecules in the solvent affect the adsorption capacity [20]. These results are shown in Table 4. With increasing pH, the adsorption capacity of NKA-9 resin increased, and the adsorption capacity of the resin peaked at pH 5. Therefore, pH 5.0 was used for the next experiment.

#### 2.7.3. Adsorption Kinetics Experiment

**Figure 4:**
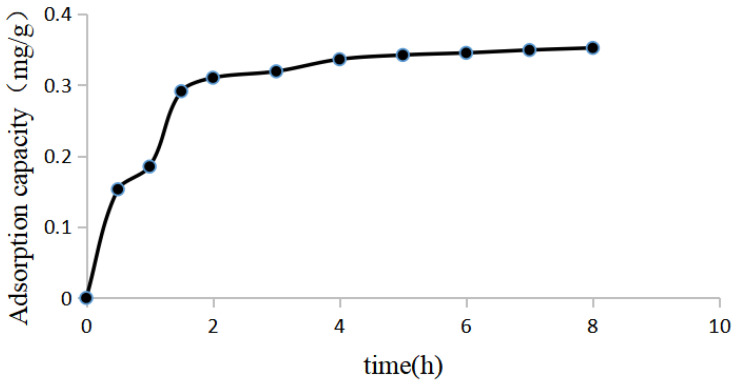
Adsorption kinetics curves of luteolin on NKA-9 resin. The results of the adsorption kinetics experiment showed that the adsorption of luteolin from *P. villosa* by NKA-9 resin reached equilibrium after 5 h of adsorption at 25 °C.

These results are shown in Figure 4. The NKA-9 resin adsorption equilibrium was reached at 5 h, after which time the luteolin in the solution was no longer adsorbed.

#### 2.7.4. Adsorption Isotherm

**Figure 5:**
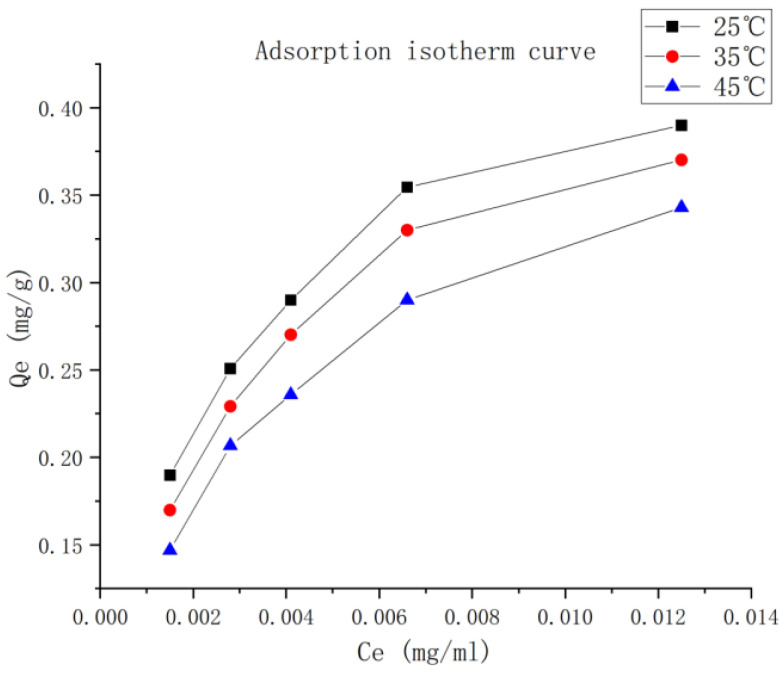
Adsorption isotherms of NKA-9 resin at different temperatures. The adsorption capacity of NKA-9 resin decreased with increasing extraction temperature, which demonstrates that the adsorption process was exothermic. This indicates that luteolin is more easily adsorbed at low temperature.

These results are shown in Figure 5. The adsorption capacity of NKA-9 resin decreased with increasing temperature, indicating that the adsorption by NKA-9 resin is an exothermic process. Therefore, 25 °C was selected for subsequent experiments. When the initial concentration of luteolin was 19.58 μg/mL, the adsorption capacity of NKA-9 resin also increased with increasing luteolin concentration until saturation was reached. Therefore, in this study, the initial concentration of luteolin was set as 19.58 μg/mL. These results are shown in Table 5. In this study, the correlation coefficients of the Langmuir equation (0.9905–0.9940) and Freundlich equation (0.9746–0.9511) were close to 1, which demonstrates that the Langmuir and Freundlich adsorption models are applicable to the process of luteolin adsorption from *P. villosa* by NKA-9 resin. Moreover, an n value of greater than 1 indicates that luteolin is adsorbed by the macroporous resin NKA-9.

#### 2.7.5. Loading Rate

These results are shown in Table 6. The faster the flow rate was, the shorter the contact time between NKA-9 and the target component, and the lower the adsorption rate of luteolin. It is necessary to consider factors such as the efficiency during the experiment. Finally, the sample loading rate for subsequent experiments was determined to be 2 BV/h.

#### 2.7.6. Penetration Curve

**Figure 6:**
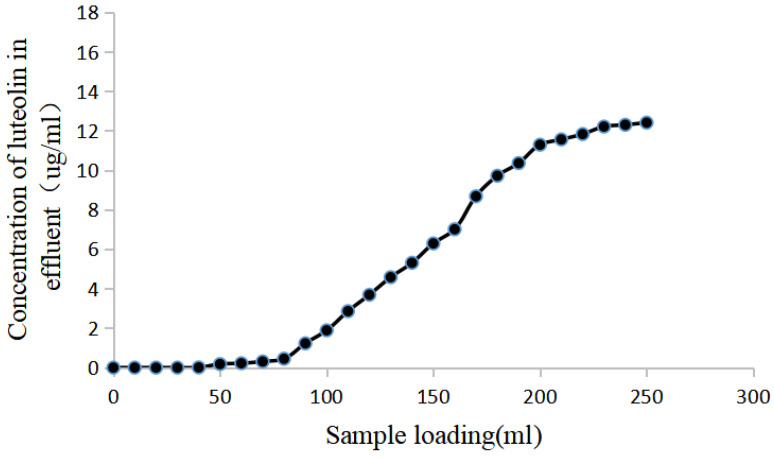
Dynamic breakthrough curves of luteolin on NKA-9 resin. The leakage point, also known as the penetration point, refers to the point at which the concentration of a target component in the effluent is 10% of that of the concentration in the sample solution after the resin reaches adsorption saturation. In this study, the saturated loading amount of luteolin sample solution on NKA-9 resin was 90 mL (2.9 BV).

As shown in Figure 6, NKA-9 resin almost completely adsorbed luteolin after 90 mL of sample was loaded, and then the concentration of luteolin in the effluent showed a significant upwards trend. Then, the concentration of luteolin in the effluent tended to stabilize after 200 mL was loaded. Therefore, 90 mL was set as the saturated loading volume in this study.

#### 2.7.7. Dynamic Gradient Elution

These results are shown in Table 7. When the ethanol concentration reached 90%, luteolin was eluted in large quantities, reaching the peak value. However, the quantity of the target eluted at ethanol concentrations of 80% and 90% was similar. Considering cost, the ethanol concentration in the eluent was finally determined to be 80%.

#### 2.7.8. Process Validation

These results are shown in Table 8. After enrichment, the luteolin content was 2.30% and the recovery rate was 80.06 ± 1.16%. This result is similar to the previous experimental results. Therefore, this model is suitable for the process of luteolin enrichment.

### 2.8. Evaluation of the Anti-PRV Activity of Luteolin-Enriched Products from P. villosa

#### 2.8.1. Anti-PRV Effects of the Luteolin-Enriched Products In Vitro

(1) Toxicity of the luteolin-enriched products to PK-15 cells

**Figure 7:**
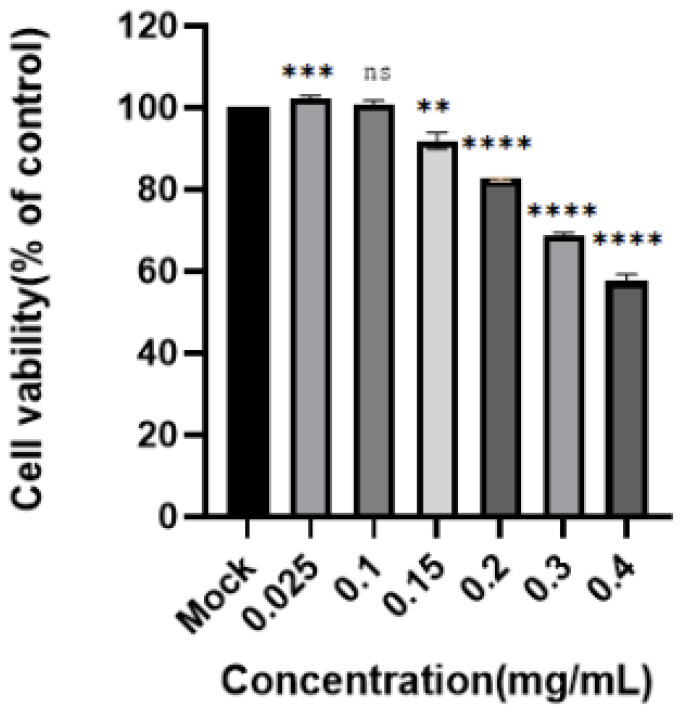
Toxicity of the enriched products to PK-15 cells. A CCK8 assay was used to determine the toxicity of DAE to PK15 cells at concentrations of 0.4, 0.3, 0.2, 0.15, 0.1, and 0.025 mg/mL. Cell viability is expressed as a percentage of control cell viability (****: *p* < 0.0001, ***: *p* < 0.001, ** *p* < 0.01; ns: *p* > 0.05).

The toxicity of the enriched product to PK-15 cells was determined by the CCK8 method. These results are shown in Figure 7. With increasing drug concentration, the cell survival rate decreased significantly. When the concentration of the enriched product was 0.15 mg/mL, no significant effect on cell proliferation was observed. According to the nonlinear regression analysis performed in GraphPad Prism 8, the CC_50_ was 0.1413 ± 0.01 mg/mL. Finally, 0.15 mg/mL of enriched product was used for the next experiment.

(2) Experimental inhibitory effect of the luteolin-enriched products on PRV

**Figure 8:**
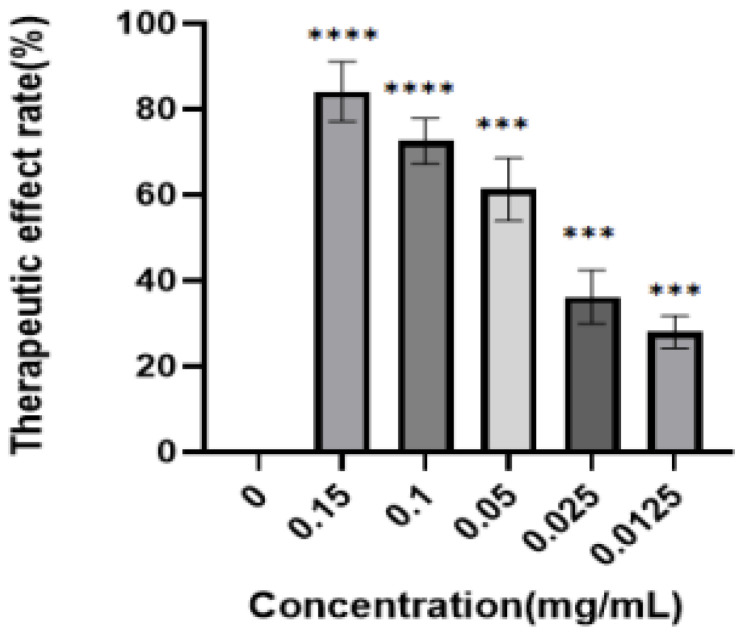
Inhibition of PRV by the enriched products. PK-15 cells were first infected with PRV and then treated with the enriched products. After 48 h, the inhibition rate was determined by the CCK8 method. The analysis showed that the inhibition rate of the luteolin-enriched product was 84.13 ± 3.22%, the IC_50_ was 0.04 ± 0.012 mg/mL, and the SI was 3.5, indicating low toxicity and high efficiency (****: *p* < 0.0001, ***: *p* < 0.001).

These results are shown in Figure 8. PK-15 cells were first infected with PRV and then treated with enriched products. After 48 h, the inhibition rate was determined by the CCK8 method. The analysis showed that the inhibition rate of the luteolin-enriched product was 84.13 ± 3.22%, the IC_50_ was 0.04 ± 0.012 mg/mL, and the SI was 3.50, indicating low toxicity and high efficiency.

#### 2.8.2. Evaluation of the Anti-PRV Effect of the Luteolin-Enriched Products In Vivo

##### Determination of the Mouse Challenge Dose

**Figure 9:**
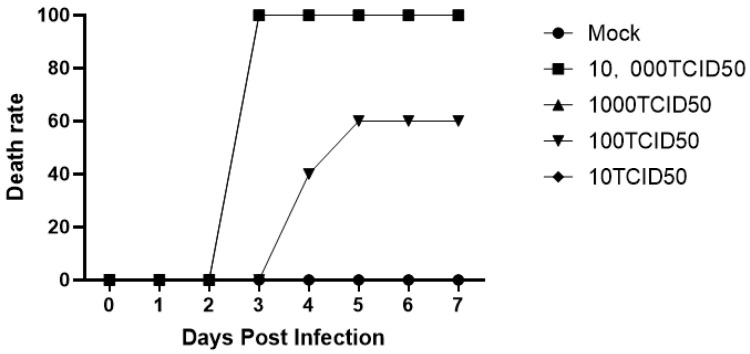
Survival rate of challenged mice. Mice were injected intramuscularly with diluted virus. The 1000 TCID_50_ group began to itch and bite the injection site frantically on the third day. The survival rate was 0. The situation of mice in the 10,000 TCID_50_ group was identical to that of mice in the 1000 TCID_50_ group. An intramuscular injection of 100 μL of 1000 TCID_50_ virus was used as the challenge dose for the mice.

The virus was diluted to a gradient of concentrations, and the virus diluent was injected into the muscles of mice. The condition of the mice was observed every day, and their mortality was calculated. These results are shown in Figure 9. The mice in the 10 TCID_50_ group showed no symptoms, and the survival rate was 100%, while the mice in the 100 TCID_50_ group exhibited obvious symptoms on the fourth day, and the survival rate was 40%. In the group intramuscularly injected with 1000 TCID_50_, itching was observed along with fervent biting at the injection site on the third day, and the survival rate was 0%. The observations of the mice in the 10,000 TCID_50_ group were identical to those of mice in the 1000 TCID_50_ group. An intramuscular injection of 100 μL of 1000 TCID_50_ virus was selected as the final challenge dose for mice.

##### Toxicity of Luteolin-Enriched Products to Mice

**Figure 10:**
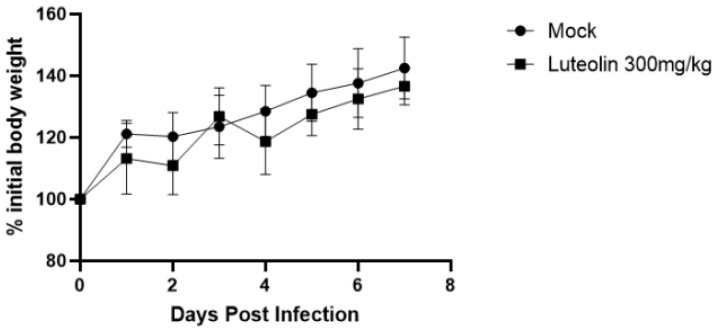
Body weight changes in the mice after administration. Compared with the normal group, the weight gain of mice in the drug group was not significantly different after the drug was injected, and no mice died. Therefore, it was decided to conduct the next test at a drug dose of 300 mg/kg.

Luteolin-enriched product (300 mg/kg) was intraperitoneally injected into mice, and the mice were observed every day and their weights were measured. These results are shown in Figure 10. Compared with that in the normal group, the weight gain of the mice in the drug group was not significantly different after drug injection, and no mice died. Therefore, it was determined that a dose of 300 mg/kg luteolin-enriched product would be used for subsequent experiments.

##### Anti-PRV Effect of the Luteolin-Enriched Products In Vivo

**Figure 11:**
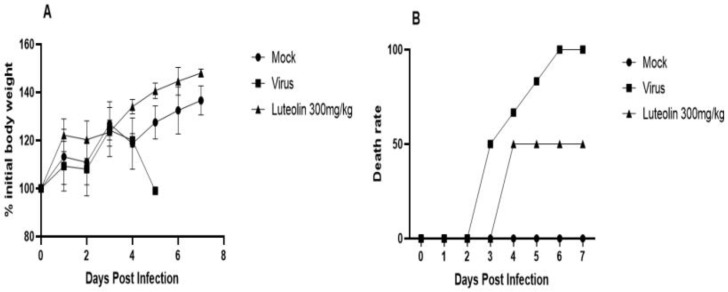
Changes in body weight and death rate of the mice treated with the drug. All the subjects in the challenge group died on the 6th day. Obvious neurological symptoms appeared on the third day. The onset time for mice in the drug group (4 days) was significantly delayed compared with that in the challenge group, and the survival rate was 50%. Compared with the normal group, the weight gain of mice in the challenge group slowed, and the weight of mice in the drug group fluctuated significantly compared with that of the normal group. (**A**) Changes in mouse body weight; (**B**) Mouse mortality rate.

These results are shown in Figure 11. In the experiment assessing the protective effect of the luteolin-enriched product on mice challenged with virus, all mice in the challenge group died on the sixth day. Obvious neurological symptoms were observed on the third day, and the mice died quickly after the onset of symptoms. The mice in the luteolin-enriched product group developed disease on the fourth day, which was significantly delayed compared with the mice in the challenge group. The survival rate of the mice in the treated group was 50%. Compared with that of the normal group, the weight gain of the mice in the challenge group slowed, and the weights of mice in the drug group fluctuated significantly compared with those in the normal group.

##### Effect of Luteolin-Enriched Products on the Viral Load in the Organs of Infected Mice

**Figure 12:**
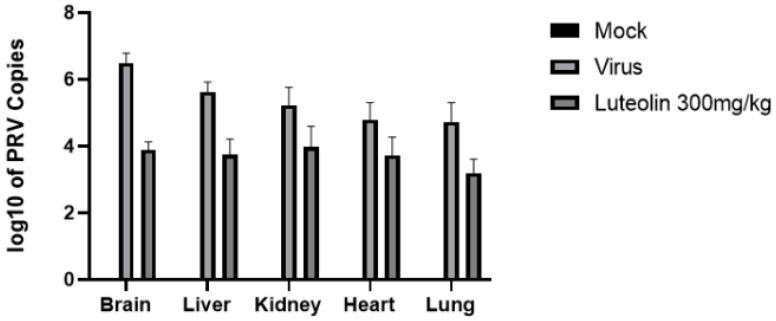
Viral loads in the organs of mice after challenge. The viral loads in the brain, liver, heart, kidney, and lung of mice were detected by FQ–PCR.

Viral DNA was collected and extracted from the liver, heart, kidney, lung, and brain tissues of the mice, and the viral load in each was evaluated by quantifying the viral content by FQ–PCR. As shown in Figure 12, the viral load in the brain tissue was the highest. Compared with that in the virus control group, the viral load in the brains of the mice in the luteolin-rich product group was significantly reduced 1.6-fold.

##### Effect of the Luteolin-Enriched Products on the Pathological Changes in Infected Mouse Brain Tissue

**Figure 13:**
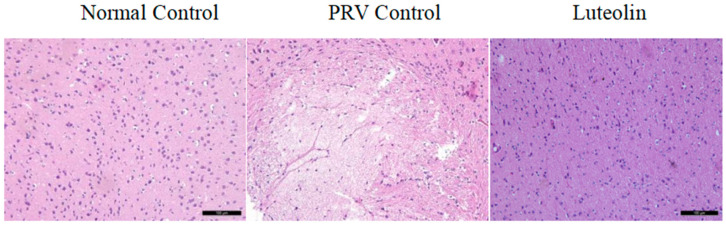
Pathological sectioning of mouse brain tissue. The brains from mice in each group were dissected for pathological evaluations, and capillary congestion in the brain tissue was found to be slowed in the drug group. The pathological changes in brain tissue in the drug group were milder than those in the challenge group, no sieve-like softening regions or capillary congestion were observed, and only a small amount of lymphocyte infiltration occurred.

The brains of mice from each group were dissected for pathological evaluations, and capillary congestion in the brain tissue was found to be reduced in the drug group. These results are shown in Figure 13. Compared with the normal group, the challenge group showed a large amount of lymphocyte infiltration, sieve-like softening lesions, neuronal degeneration, and necrosis in the brain tissues. The pathological changes in the drug group were less severe than those in the challenge group. No sieve-like softening lesions or capillary congestion were observed, although there was a small amount of lymphocyte infiltration.

## 3. Discussion

Hot reflux extraction is widely used in laboratories and industrial production because this method is simple to perform, the energy consumption is low, and the extraction rate is high. This method uses volatile organic solvents to extract target components, and the evaporated solvent is cooled in the container for further extraction until the target components are completely extracted. At present, the hot reflux extraction method has been successfully applied to extract various effective components from plants. The BBD experimental response surface methodology was invented by Behnken and Box in 1960. This method can continuously analyze all experimental levels while optimizing the experimental conditions and can also be used to solve problems related to nonlinear data processing. This method can establish mathematical models, check the suitability of models, determine the best combination of conditions, and be used for many other experiments. It is widely used to optimize extraction processes. On the basis of a single-factor experiment, the process of extracting luteolin from *P. villosa* was optimized by a BBD response surface methodology. The optimum extraction conditions were determined as follows: an extraction time of 43.00 min, a methanol/hydrochloric acid solvent ratio of 13:1, an extraction temperature of 77.60 °C, a material-to-liquid ratio of 1:22, and two extractions. Under the optimized conditions, the extraction rate of luteolin was 0.265 ± 0.03 mg/g, which was close to the theoretical value. This indicated that the extraction process optimized by the response surface methodology was suitable for extracting luteolin from *P. villosa* and was stable and feasible.

Macroporous resin can adsorb molecules by participating in van der Waals or hydrogen bonding interactions with the target molecules. Due to their advantages, such as fast adsorption, large capacity, stable chemical properties, and reduced energy consumption, resins are applicable in various fields. This approach can approximately classify target compounds or the effective components in traditional Chinese medicines. The adsorption isotherm shows that the adsorption capacity of NKA-9 resin decreased with increasing extraction temperature, which demonstrates that the adsorption process was exothermic. The optimized adsorption conditions were a pH of 5.0, a temperature of 25 °C, an initial concentration of luteolin of 19.58 µg/mL, a sample loading volume of 2.9 BV, and a sample loading speed of 2 BV/h. The optimized desorption conditions were distilled water, 30% ethanol and 80% ethanol elution, and 5 BV at a flow rate of 2 BV/h. Under the optimized conditions, the recovery rate of enriched luteolin reached 80.06%, and its content increased 3.4-fold. This result indicated that NKA-9 resin has the ability to enrich luteolin, providing scientific data support for the preparation of luteolin and further research on its efficacy.

The antiviral mechanisms of traditional Chinese medicines are generally divided into the following categories: In one category, the agent directly inhibits the activity or replication of the virus, including by blocking virus adsorption or entry, inhibiting virus synthesis, or blocking viral mRNA replication or protein synthesis. In the second category, the body’s immune system is regulated to indirectly resist viruses. In recent years, studies have confirmed that the total flavonoids in *P. villosa* have obvious antiviral effects [21,22], and luteolin is an important flavonoid compound that exists in many plants. Previous results showed that the 3′,4′-OH and 5′-OH groups in the chemical structure of luteolin were the main moieties closely related to antiviral activity. At present, few studies have evaluated the antiviral activity of luteolin from *P. villosa*. Therefore, this experiment preliminarily evaluated the anti-PRV activity of luteolin-enriched products from *P. villosa* in vitro and in vivo. PRV is a DNA virus in the family Herpesviridae that has a relatively high infection rate in pigs. It is difficult to thoroughly purify samples from infected pigs [23], and these infections can cause strong disease symptoms and persistent high fever. The virus can also cause abortion in sows, and it has been reported that encephalitis can occur in humans after infection [24]. Therefore, it is of great public health significance to research methods for preventing and controlling PRV. The invasion of PRV into host cells mainly involves adsorption, entry, replication, maturation, and other processes. Drugs can inhibit any of these processes [25]. In this study, infected PK-15 cells were used as an in vitro model to assess the anti-PRV effect of the luteolin-enriched products, and the prevention, treatment, and direct effects of luteolin-enriched products on PRV were studied. The results showed that the treatment effect was substantial, as the luteolin-enriched products could enter cells and inhibit the viruses in cells; the inhibition rate was 84.13 ± 5.22%, the IC_50_ was 0.04 ± 0.012 mg/mL, and the SI was 3.5, indicating low toxicity and high efficiency. The in vivo experiment closely resembled clinical conditions, and the results can be used for comprehensive assessment. In this study, mice infected with PRV were used as animal models to assess the anti-PRV effect of the luteolin-enriched products. The luteolin-enriched products delayed the onset of PRV infection in mice and improved the survival rate of infected mice. PRV mainly attacks the nervous system, so brain tissue was selected for analysis, and pathological changes were observed in brain tissue. The mice in the virus group exhibited obvious lymphocyte infiltration, sieve-like softening lesions, and a large amount of neuronal degeneration and necrosis, while the luteolin-enriched products reduced the damage to the brain tissue caused by the virus. Capillary congestion was also significantly reduced, which was consistent with the research of Li et al. [26]. The luteolin-enriched products showed obvious inhibitory effects in the preliminary anti-PRV activity in the in vitro and in vivo experiments, which provides supporting scientific data for the research and development of anti-PRV traditional Chinese medicines.

## 4. Materials and Methods

### 4.1. Materials and Instruments

A whole plant of *P. villosa* (Herba Patriniae) was collected from Lixian County, Longnan City, China (N 33°35′31′′, E 104°37′36′′), in October 2020 and was authenticated by Professor Yan Zhu (Northeast Agricultural University). The plant was stored in the Key Laboratory of Animal Disease Control and Drug Development of Northeast Agricultural University. Luteolin was purchased from Dalian Meilun Biotechnology Co., Ltd. (Dalian, China) Formic acid (chromatographically pure) and acetonitrile (chromatographically pure) were obtained from Tianjin Kemio Chemical Reagent Co., Ltd. (Tianjin, China) Anhydrous methanol and ethanol were purchased from Tianjin Fuyu Fine Chemical Co., Ltd. (Tianjin, China) PK-15 cells and the PRV TJ strain were provided by Harbin Institute of Veterinary Medicine, Chinese Academy of Agricultural Sciences. An LC-10A high-performance liquid chromatograph (Shimadzu International Trade Co., Ltd., Shanghai, China) was used for the analysis of luteolin in *P. villosa*. A rotary evaporator (Shanghai Yarong Biochemical Instrument Factory, Shanghai, China), CO_2_ cell incubator (HF151), and automatic enzyme label detector (ELx808, Betheng Instrument Co., Ltd., Rockville, MD, USA) were also used.

### 4.2. Extraction of Luteolin

A total of 2.00 g of screened *P. villosa* sample was accurately weighed, and then hot reflux extraction was performed with a preset extraction time, extraction temperature, material/liquid ratio, and methanol/hydrochloric acid solvent ratio, and preset centrifugation parameters (3000 r/min, 5 min). The sample was passed through a 5 μm filter membrane and stored until subsequent analysis.

### 4.3. Determination of the Luteolin Content

The content of luteolin was determined by chromatography under the following conditions: sample volume, 0.02 mL; column temperature, 25 °C; detection wavelength, 350 nm; mobile phase, 0.1% formic acid (A)/acetonitrile (B) with gradient elution (0~0.01 min, 5% B → 1% B; 0.01~37.50 min, 1% B → 100% B; 37.50~45 min, 100% B → 90% B; and 45~50 min, 90% B → 5% B); and flow rate, 1 mL/min. The equation of the standard calibration curve was y = 5 × 108x + 13763, where x is the sample concentration in the range of 0.0288–0.00288 mg/mL and y is the peak area (R^2^ = 0.9995).

### 4.4. Experimental Design and Statistical Analysis

The results of the single-factor experiment were analyzed using SPSS 11.5 software (SPSS Inc., Chicago, IL, USA). The orthogonal experiment was designed using Design Expert 8.05b software, and the data obtained were analyzed by regression. Many parameters affect the extraction rate of luteolin; therefore, not all factors could be determined, and only the main factors were selected. After consulting a large number of studies, the extraction time (15 min, 30 min, 45 min, 60 min, and 75 min), extraction temperature (40 °C, 50 °C, 60 °C, 70 °C, and 80 °C), material/liquid ratio (1:10, 1:15, 1:20, 1:25, and 1:30), hydrochloric acid/methanol solvent ratio (1:5, 1:10, 1:15, 1:20, and 1:25), and number of extractions (1, 2, 3, 4, and 5 times) were selected for single-factor experiments. Then, the response surface method was used to design the experiments. As shown in Table 9, experiments design based on response surface methodology were conducted. In this study, the following factors were selected: extraction time, material/liquid ratio, extraction temperature, and methanol/hydrochloric acid solvent ratio. These factors were defined as X_1_, X_2_, X_3_, and X_4_, respectively. The low, medium, and high levels were coded as—1, 0, and 1, respectively, and the data are expressed as the mean ± standard deviation (SD). A second-order polynomial model was used to describe the relationship between the luteolin extraction rate (response value) and various factors (independent variables), and the optimal extraction parameters were predicted on the basis of fitting the data and analysis of variance (ANOVA).

### 4.5. Validation Experiment

Under the optimal extraction conditions, luteolin was extracted from *P. villosa* by hot reflux, and the extraction rate was calculated. The difference between the calculated value and the extraction rate predicted by response surface methodology was analyzed to verify whether the experimental value was consistent with the prediction.

### 4.6. Luteolin Enrichment

#### 4.6.1. Pretreatment of the Macroporous Resin

The macroporous resins NKA-9, X-5, D-101, HPD-100, HPD-300, HPD-826, HPD-600, and HPD-400 were soaked in 95% ethanol for 24 h and then washed continuously with distilled water until the effluent exhibited neither traces of alcohol nor turbidity. The physical properties of these resins are summarized in Table 10.

#### 4.6.2. Static Adsorption

(1) Adsorption and desorption

A total of 0.30 g of pretreated resin was weighed and placed into a conical flask. Then, 10 mL of luteolin solution was added to each conical flask, which were placed in a constant-temperature shaking incubator at 25 °C and shaken for 24 h at 120 revolutions per minute. Then, the impurities were washed away with 50 mL of distilled water. After filtering and draining each macroporous resin, 50 mL of 70% ethanol solution was added, and the mixture was shaken for 24 h under the same conditions. The contents of luteolin in the adsorbed solution and desorbed solution were determined by HPLC. The amount adsorbed, amount desorbed, and desorption rate of each macroporous resin were calculated according to the formulas below, and the experiment was repeated three times for each resin. The adsorption capacity, desorption capacity, and desorption rate were used to establish a resin model for subsequent experiments. The calculation formulas are as follows: Qe = (C_0_ – C_e_)V_0_/W, Q_d_ = V_d_C_d_/W, and D = V_d_C_d_/(C_0_ – C_e_)V_0_ × 100%, where Q_d_ is the amount desorbed per gram of resin (mg/g); Q_e_ is the amount adsorbed per gram of dry resin (mg/g); C_0_ is the initial concentration (mg/mL); C_e_ is the concentration after adsorption equilibrium (mg/mL); C_d_ is the concentration of desorption solution (mg/mL); V_0_ is the initial volume of the sample (mL); V_d_ is the volume of desorption solution (mL); W is the dry weight of the resin (g); and D is the desorption rate (%).

(2) Effect of pH on the adsorption capacity of the macroporous resins

Three 10 mL luteolin solutions were placed into conical flasks. After the pH values were adjusted to 3.00, 5.00, and 7.00, 0.50 g of NKA-9 macroporous resin was accurately weighed and added. The samples were placed in a constant-temperature shaking incubator at 25 °C and shaken at 120 r/min for 6 h. After reaching adsorption equilibrium, the solution was extracted, and the luteolin content was measured by HPLC to calculate the adsorption capacity of the macroporous resin.

(3) Adsorption kinetics

After accurately weighing 2.00 g of NKA-9 resin, 20 mL of luteolin sample solution was added, and the sample was placed in a constant-temperature shaking incubator at 120 r/min and 25 °C and shaken for 0.50, 1, 1.50, 2, 2.50, 3, 4, 5, 6, 7, or 8 h. The luteolin content was determined, the amount adsorbed was calculated, and the kinetic curve of luteolin adsorption by NKA-9 was drawn.

(4) Static adsorption isotherm

Five 1.00 g portions of NKA-9 macroporous resin were weighed, and 10 mL aliquots of different concentrations of the luteolin sample solution were added. Then, the samples were placed in a constant-temperature shaking incubator at 25, 35, and 45 °C and shaken at 120 r/min for 5 h. The content of luteolin was determined, and the adsorption capacity of the resin was calculated for each sample with a different concentration of luteolin. The fit to the Freundlich and Langmuir isotherm adsorption model was evaluated based on the relationship between the concentration of luteolin in solution after equilibrium and the adsorption capacity of the resin after equilibrium. The adsorption isotherm was drawn, and the relevant parameters were calculated. The Freundlich isotherm adsorption equation is as follows: Q_e_ = K_F_C_e_1/n.

The Langmuir isotherm adsorption equation is as follows: Q_e_ = Q_m_ K_L_C_e_/(1 + K_L_C_e_), where Q_m_ is the amount adsorbed at saturation (mg/g); C_e_ is the concentration in solution after equilibrium (mg/mL); Q_e_ is the amount adsorbed at equilibrium (mg/g); K_L_ is the Langmuir adsorption equilibrium constant (mL/mg); and n represents the change trend of the isotherm. K_F_ is the characteristic constant of the Freundlich adsorption isotherm (mL/mg), and K_L_ represents the amount adsorbed.

#### 4.6.3. Dynamic Adsorption

(1) Sample loading rate

A 15 mm i.d. glass chromatographic column was filled with 30 mL of prewetted NKA-9 resin to 400 mm, and 5 BV sample solutions were added at different loading rates (1, 2, and 4 BV/h). After reaching adsorption equilibrium, the content of luteolin in the effluent was determined, and then the column was eluted with 5 BV distilled water and 5 BV 70% ethanol. The content of luteolin in the water and 70% ethanol washing solutions was determined, and the amount of luteolin adsorbed on the NKA-9 resin was calculated as follows to determine the optimal loading rate: Q = M_upper_ − M_residue_ − M_water washing_. Here, M_water washing_ is the mass of luteolin after washing (mg); Q is the amount adsorbed (mg/g); M_residue_ is the mass of luteolin in the column effluent (mg); and M_upper_ is the mass of luteolin in the upper column solution (mg).

(2) Breakthrough curve

Luteolin solution (5 BV) was added to 400 mm × 30 mL of prewetted NKA-9 resin filled in a 15 mm i.d. glass chromatographic column with a sample loading rate of 2 BV/h. Ten milliliter fractions were collected and the concentration of luteolin was measured in each to construct a breakthrough curve.

(3) Eluent concentration determination

First, 400 mm × 30 mL of prewetted NKA-9 resin was added to fill a 15 mm i.d. glass chromatographic column with a sample loading rate of 2 BV/h. After the resin was saturated, the sample was first eluted with 5 BV distilled water and then eluted with 5 BV of a sequence of 10%, 20%, 40%, 60%, 80%, and 90% ethanol. The elution rate was 2 BV/h. The desorption capacity of the luteolin from the analyzed resin was determined.

#### 4.6.4. Process Validation

A 7.50 cm i.d. resin column was filled to 100 cm with prewetted NKA-9. The optimal process conditions were obtained by calculations, and the results under these conditions were verified. The experiment was repeated three times. Finally, the adsorption conditions were determined as follows: the adsorption temperature was kept at 25 °C, the initial concentration of luteolin was 19.58 µg/mL, the pH value was 5, the sample loading volume was 2.90 BV, and the sample loading rate was 2 BV/h. During desorption, distilled water, 30% ethanol, and 80% ethanol were used successively to elute the sample. The elution rate was 2 BV/h, and the elution amount was 5 BV.

### 4.7. Evaluation of the Anti-PRV Effect of the Luteolin-Enriched Products from P. villosa

#### 4.7.1. Determination of the Anti-PRV Effects of the Luteolin-Enriched Products In Vitro

(1) Determination of the toxicity of luteolin-enriched products to PK-15 cells

The luteolin-enriched product was accurately weighed, dissolved in maintenance solution, and serially diluted to 6 different concentrations. Each concentration was evaluated 3 times, and each experiment was repeated 3 times. A cell control was used. PK-15 cells were cultured at 37 °C under 5% CO_2_. PK-15 cells were subcultured in 96-well plates at a ratio of 1:6 prior to the experiment. When the cells grew to approximately 80% confluence, the discarded solution was observed under the microscope, and the cells were washed three times with PBS. Concentrated product diluent was added to 200 µL, and 200 µL of maintenance solution was added to the cell control. After culture in a cell incubator for 48 h at 37 °C under 5% CO_2_, the culture solution was discarded. Washing with PBS was performed 2–3 times. In total, 10 µL of CCK8 and 100 µL of cell maintenance solution were added to each well, and the plates were placed at 37 °C in a 5% CO_2_ incubator for 30–60 min. Then, the OD value was measured at a wavelength of 450 nm. The cell survival rate was calculated according to the following formula, and the CC_50_ values were subjected to nonlinear regression analysis with GraphPad.

Cell survival rate (%) = (average OD value of the drug group/average OD value of the cell control group) × 100%.

(2) Inhibition of PRV by the luteolin-enriched products

Viral diluent (100 TCID_50_) was added to each well (100 µL), and the samples were incubated at 37 °C in a 5% CO_2_ incubator for 1 h. Incompletely adsorbed virus diluent was discarded, and the wells were washed with PBS 2–3 times. Then, the enriched product was diluted with 100 μL, and culture was continued in the incubator for 48 h. The culture medium was then discarded, and the cells were washed with PBS 2–3 times. In total, 10 µL of CCK8 and 100 µL of cell maintenance solution were added to each well, and the samples were incubated in a 37 °C, 5% CO_2_ incubator for 30 min to 60 min. The OD values were measured at 450 nm. The inactivation rate was calculated and analyzed, and nonlinear regression was performed with GraphPad to determine the IC_50_ value and calculate the selectivity index (SI). SI = CC_50_/IC_50_ (an SI > 2 indicates that the drug has low toxicity and is highly effective; an SI between 1 and 2 indicates that the drug is highly toxic and not very effective; and an SI < 1 indicates that the drug is invalid). Inactivation rate (%) = [(average OD value of the drug group − average OD value of the virus control group)/(average OD value of the cell control group − average OD value of the virus control group)] × 100%.

#### 4.7.2. Determination of the Anti-PRV Effect of the Luteolin-Enriched Products In Vivo

Eighteen 5-week-old female Kunming mice were purchased from the Longevity Experimental Animal Center and randomly divided into the following groups in the Experimental Animal Laboratory of Northeast Agricultural University: normal group, luteolin-enriched product group (300 mg/kg), and challenge group. A total of 1000 TCID_50_ PRV was intramuscularly injected into the enriched product group and the challenge group, and DMEM was intramuscularly injected into the normal control group. One hour later, DMEM was intraperitoneally injected into the challenge group and the normal group. The enriched product group was treated for three consecutive days. The weights and symptoms of the mice were observed every day, and the mortality was calculated. Three mice were randomly selected from the 300 mg/kg luteolin-enriched product group and the control group to obtain tissues and organs (liver, kidney, heart, lung, and brain) for dissection. Samples of the heart, liver, lung, kidney, and brain were collected from each group and homogenized to extract DNA. The viral load was determined by FQ–PCR. The remaining mice were monitored to determine survival during the remaining experimental period. Immediately after death, the mice were dissected and the pathological changes in the eye and brain were recorded. The brain tissue was fixed in 4% formaldehyde, and then histological sectioning and pathological examinations were performed.

## 5. Conclusions

1. The extraction and enrichment processes of Luteolin from Patrinia villosa were optimized.
2. The enriched product of Luteolin from Patrinia villosa has anti-PRV activity in vivo and in vitro.

## Figures and Tables

**Table 1:** Experimental parameters of the Box–Behnken design and results.

Number	X_1_ (Time)	X_2_ (Liquid/Material Ratio)	(X_3_) (Temperature)	X_4_ (Solvent Ratio)	Y (Extraction Rate, mg/g)
1	45	25	70	15	0.241
2	45	20	60	15	0.228
3	45	20	70	20	0.1936
4	45	20	80	15	0.2584
5	45	15	70	15	0.2001
6	45	20	70	10	0.2586
7	30	20	70	15	0.1712
8	30	20	70	15	0.1774
9	30	20	60	10	0.1766
10	30	20	60	20	0.1532
11	30	20	70	15	0.1778
12	30	20	80	20	0.1668
13	30	15	70	20	0.1622
14	30	25	70	10	0.2016
15	30	25	70	20	0.1795
16	30	20	80	10	0.257
17	30	15	80	15	0.1776
18	30	15	70	10	0.208
19	30	25	80	15	0.2272
20	30	25	60	15	0.199
21	30	15	60	15	0.1528
22	15	15	70	15	0.1238
23	15	20	80	15	0.1628
24	15	25	70	15	0.1378
25	15	20	60	15	0.1363
26	15	20	70	20	0.1128
27	15	20	70	10	0.1522

**Table 2:** ANOVA of the response surface quadratic model analysis of variance.

Source	Sum of Squares	Degrees of Freedom	Mean Square	F Value	*p*-Value (Prob > F)	
Model	0.0406	14	0.0029	17.98	<0.0001	significant
X_1_	0.0256	1	0.0256	158.6	<0.0001	
X_2_	0.0022	1	0.0022	13.5	0.0032	
X_3_	0.0035	1	0.0035	21.48	0.0006	
X_4_	0.0068	1	0.0068	42.24	<0.0001	
X_1_X_2_	0.0002	1	0.0002	1.12	0.3104	
X_1_X_3_	3.80 × 10^−6^	1	3.80 × 10^−6^	0.0236	0.8805	
X_1_X_4_	0.0002	1	0.0002	1.02	0.3334	
X_2_X_3_	2.89 × 10^−6^	1	2.89 × 10^−6^	0.0179	0.8957	
X_2_X_4_	0.0001	1	0.0001	0.8708	0.3691	
X_3_X_4_	0.0011	1	0.0011	6.92	0.022	
X_1_^2^	0	1	0	0.1097	0.7463	
X_2_^2^	0	1	0	0.2019	0.6612	
X_3_^2^	0.0009	1	0.0009	5.68	0.0345	
X_4_^2^	0.0001	1	0.0001	0.5052	0.4908	
R^2^	0.0019	12	0.0002			
Adj-R^2^	0.0019	10	0.0002	13.93	0.0688	not significant
Pred-R^2^	0.0002	2	0.0001			
Adequate precision	0.0425	26				

**Table 3:** Predicted and actual experimental values under the optimized conditions.

Optimum Condition				Extraction Rate (mg/g)	
Extraction Time (min)	Extraction Temperature (°C)	Solvent Ratio (Methanol/Hydrochloric Acid)	Liquid/Material Ratio (mL/g)	Actual Value	Predicted Value
43	77.6	13	22	0.265 ± 0.03	0.261

**Table 4:** Effect of pH on the adsorption capacity of luteolin by NKA-9.

pH Value	Luteolin Adsorption Capacity (mg/g)
3	0.18
5	0.2
7	0.14

**Table 5:** Langmuir and Freundlich adsorption parameters at 25, 35 and 45 °C.

Temperature	Langmuir Equation	R^2^	Qm	Freundlich Equation	R^2^	1/n
(°C)			(mg/g)			
25	Ce/Qe = 2.1492Ce + 0.0049	0.99049	0.47	Qe = 1.85Ce^0.3765^	0.97456	0.3765
35	Ce/Qe = 2.2138Ce + 0.0056	0.99516	0.45	Qe = 1.76Ce^0.3366^	0.94503	0.3366
45	Ce/Qe = 2.3612Ce + 0.0071	0.994	0.42	Qe = 1.69Ce^0.3244^	0.95114	0.3244

**Table 6:** Effect of flow rate on the adsorption capacity of NKA-9 resin.

Flow Rate (BV/h)	Luteolin Adsorption Rate (%)
1	87.60%
2	85.70%
4	80.20%

**Table 7:** Results of gradient elution of luteolin from NKA-9 resin.

Ethanol Concentration (%)	Luteolin Content (mg)
0%	0
10%	0.0428
20%	0.0756
40%	0.0948
60%	0.1789
80%	0.2231
90%	0.2393

**Table 8:** Purification result of luteolin on column by NKA-9 resin under optimized conditions.

Treatment Condition	Solid Mass (g)	Luteolin Content (%)	Rate of Recovery (%)
Crude extract	50	0.60%	80.06% ± 1.16
80% ethanol concentrate	11.62 ± 0.23	2.30%	

**Table 9:** Variables and experimental levels of the response surface design.

		Level		
Variable	Code	−1	0	1
Extraction time (min)	X1	15	30	45
Material/liquid ratio (mg/mL)	X2	15	20	25
Extraction temperature (°C)	X3	60	70	80
Solvent ratio (%)	X4	10:01	15:01	20:01

**Table 10:** Physical properties of the 8 kinds of macroporous resins.

Name	Polarity	Surface Area (m²/g)	Average Pore Diameter (nm)	Particle Diameter (mm)
NKA-9	Polar	250–290	15.5–16.5	0.30–1.25
X-5	Nonpolar	500–600	29.0–30.0	0.30–1.25
D-101	Nonpolar	480–520	25.0–28.0	0.30–1.25
HPD-100	Nonpolar	650–700	8.5–9.0	0.30–1.25
HPD-300	Moderately polar	800–870	5.0–5.5	0.30–1.20
HPD-826	Moderately polar	500–600	9.0–10.0	0.30–1.25
HPD-600	Polar	550–600	8	0.30–1.20
HPD-400	Moderately polar	500–550	7.5–8.0	0.30–1.20

## Data Availability

The original contributions presented in the study are included in the article, and further inquiries can be directed to the corresponding author.

## Sample Availability

Samples of the compounds are not available from the authors.
